# Investigation of Hybrid Electrodes of Polyaniline and Reduced Graphene Oxide with Bio-Waste-Derived Activated Carbon for Supercapacitor Applications

**DOI:** 10.3390/polym16030421

**Published:** 2024-02-02

**Authors:** Imen Benchikh, Abdelrahman Osama Ezzat, Lilia Sabantina, Youcef Benmimoun, Abdelghani Benyoucef

**Affiliations:** 1 Faculty of Science, University of Amar Telidji Laghouat, Laghouat 03000, Algeria; i.benchikh@lagh-univ.com; 2 Department of Chemistry, College of Sciences, King Saud University, Riyadh 11451, Saudi Arabia; aezzat@ksu.edu.sa; 3 Department of Apparel Engineering and Textile Processing, Berlin University of Applied Sciences-HTW Berlin, 12459 Berlin, Germany; 4 Department of Textile and Paper Engineering, Polytechnic University of Valencia (UPV), 03801 Alcoy, Spain; 5 Water Science and Technology Laboratory, University of Mustapha Stambouli Mascara, Mascara 29000, Algeria; youcef.benmimoun@univ-mascara.dz

**Keywords:** bio-waste, activated carbon, reduced graphene oxide, polyaniline, supercapacitor

## Abstract

Graphene-based materials have been widely studied in the field of supercapacitors. However, their electrochemical properties and applications are still restricted by the susceptibility of graphene-based materials to curling and agglomeration during production. This study introduces a facile method for synthesizing reduced graphene oxide (rGO) nanosheets and activated carbon based on olive stones (OS) with polyaniline (PAni) surface decoration for the development of supercapacitors. Several advanced techniques were used to examine the structural properties of the samples. The obtained PAni@OS−rGO (1:1) electrode exhibits a high electrochemical capacity of 582.6 F·g^−1^ at a current density of 0.1 A·g^−1^, and an energy density of 26.82 Wh·kg^−1^; thus, it demonstrates potential for efficacious energy storage. In addition, this electrode material exhibits remarkable cycling stability, retaining over 90.07% capacitance loss after 3000 cycles, indicating a promising long cycle life. Overall, this research highlights the potential of biomass-derived OS in the presence of PAni and rGO to advance the development of high-performance supercapacitors.

## 1. Introduction

Energy plays a crucial and pivotal role in various aspects of life, the economy and technology in today’s society; however, excessive consumption of fossil fuels requires a transition to cleaner and more sustainable energy sources such as renewable energy, as well as improvements in energy efficiency. This transition is crucial for mitigating climate change, protecting ecosystems and promoting a more sustainable and equitable future [1,2,3,4,5]. However, stable energy storage technologies are a key enabler for achieving universal access to clean energy by addressing the intermittent nature of certain renewable sources; moreover, such technologies help to provide a reliable and continuous energy supply. The development and deployment of advanced energy storage solutions are critical components of the transition to a more sustainable and resilient energy system.

Supercapacitors (SCs), also known as electrochemical capacitors, have attracted significant interest in recent years due to their unique properties and potential applications compared to Li-ion batteries [6,7,8]. The selection and design of electrode materials are fundamental in advancing the electrochemical properties of energy storage and conversion devices, influencing their efficiency, durability and overall effectiveness in various applications [9,10,11]. Moreover, the choice of electrode materials affects parameters such as the energy density, power density, cycle life and overall efficiency [12,13,14,15].

According to the electrode materials employed, SCs can be classified into three main kinds: electric double-layer capacitors (EDLCs), pseudocapacitors (PCs) and asymmetric supercapacitors (ASCs) [16,17]. EDLCs store energy through ion adsorption–desorption at the electrode–electrolyte interface, wherein the electrode predominantly comprises carbon-based materials, encompassing carbon quantum dots, carbon fibers, graphene and activated carbon [18,19,20,21]. The major advantage of EDLCs is their decent cycling stability, but their poor capacitance leads to lower energy densities (<10 Wh·kg^−1^) compared to LIBs (50–1000 Wh·kg^−1^) [22]. Conductive polymers (PCs) store energy based on rapid and reversible oxidation–reduction reactions, and electrode materials for PCs mainly include noble metals (e.g., Pt, Au, etc.), transition metal compounds (e.g., TiO_2_, TiC, ZrO_2_, Nb_2_O_5_, RuO_2_, etc.), alongside CPs such as polyaniline (PAni), polypyrrole (PPy) and polythiophene (PTh) [7,8,9,23]. The capacitance of pseudocapacitors is generally better than EDLCs, but their limited cycling stability poses challenges for long-term operation. ASCs consist of two types: capacitor/capacitor and battery/capacitor (hybrid capacitor) supercapacitors, whose greatest advantage is to utilize the potential difference between anodes and cathodes to extend their voltage window while taking into account their high capacitance, thus further enhancing their energy density to satisfy practical requirements.

Graphene and its derivatives have shown great promise in enhancing the performance of SCs, especially those based on CPs. Graphene is a single layer of carbon atoms arranged in a hexagonal lattice; it possesses exceptional electrical, mechanical and thermal properties. When incorporated into the structure of CPs, graphene can address several challenges associated with traditional materials used in SCs [7,20]. Likewise, the combination of CPs and reduced graphene oxide (rGO) in electrode materials often leads to synergistic effects that improve the overall performance of SCs [23].

Cheng et al. prepared a PAni/graphene electrode via an in situ anodic electropolymerization of PAni film on graphene paper with an admirable electrochemical capacitance of 233 F·g^–1^ [24]. The PAni/graphene electrodes with an EDLC of graphene nanosheets and a PSC of PAni presents a synergistic impact with good specific capacitance (Csp) (375.2 F·g^–1^) for flexible film supercapacitors [25]. However, the Csp in graphene/PAni electrodes provided by the graphene sheets is lower due to its agglomerated layer-like structure, and is mainly dominated from the PAni films coated on the graphene sheets [26]. Therefore, it is significant to study the electrical and electrochemical characteristics of functionalized graphene to prevent its reaggregation [25,26]. Moreover, a similar comparison can be seen in Table S1 for hybrid electrodes used for in situ polymerization with PAni and graphene.

Commercial activated carbon (AC) has been examined as electrode material in SCs; however, it is created from fossil fuels, which are not environmentally friendly, and are expensive. An emerging strategy is to develop AC electrode materials using naturally abundant bio-waste products. These bio-wastes comprise carbon-rich organic matter, so they can be a perfect feedstock for the preparation of AC. Various bio-wastes, including soyabean, coconut shell, egg shell, bamboo, dead neem leaves, banana peel and so forth, have been intensively applied to generate highly porous AC for testing in SCs [27,28,29].

Significant progress has been achieved in the field of PAni and PAni@rGO materials, and the specific capacitance and long-term cycling performance of PAni@rGO electrodes have been greatly enhanced. However, there are still some challenges with PAni@rGO composite electrodes, such as the high cost of rGO and the design of high-performance rGO frameworks. In order to solve these problems, we present a strategy to develop PAni@rGO electrodes for high-performance supercapacitors via the preparation of ternary composite. Hence, the porous AC was prepared from olive stones (OS) through a process known as carbonization [30,31]; it was subsequently used to cover rGO sheets via a simple method. Then, the OS−rGO was coated with a polymer matrix using chemical polymerization of an Ani monomer. The electrochemical performance of the prepared PAni@OS−rGO electrode-based SCs were tested. Their unique combination of high power density, fast charge/discharge rates and long cycle life makes these electrodes well-suited for applications that require rapid and reliable energy storage and release.

## 2. Materials and Methods

### 2.1. Materials and Reagents

Olives were purchased from local supermarkets in Mascara, Algeria. The following materials were acquired: graphite powder (Superior Graphite. Co.; Chicago, IL, USA, 99.9%), aniline (Ani; Aldrich; Madrid, Spain, ≥ 99.5%), ammonium persulfate (APS; Merck; Riga, Lithuania, ≥98%), polyvinylidene fluoride (PVDF), ammonium hydroxide (NH_4_OH; Merck; Riga, Lithuania, 25%), N-methylpyrrolidone (NMP), carbon black (CB, Superior Graphite. Co.; Chicago, IL, USA), sodium hydroxide (NaOH, Merck; Riga, Lithuania, 37%), sulfuric acid (H_2_SO_4_, Merck; Riga, Lithuania, 90%), hydrogen peroxide (H_2_O_2_, Merck; Riga, Lithuania, 70%), sodium nitrate (NaNO_3_), potassium hydroxide (KOH), potassium permanganate (KMnO_4_), acetone (C₃H₆O, 90%), ethanol (C_2_H_5_OH, 96%), distilled water and filter paper. A stainless steel lamina (SS; thickness of 0.2 mm) was applied as the electrode foil.

### 2.2. Apparatus

The crystalline structure of the samples was measured using an X-ray diffractometer (XRD) (CCD Apex Bruker, Madison, WI, USA). The elemental composition was investigated with an X-ray photoelectron spectrometer (XPS) (AVG Microtech Multilab, 3000 electron, Tokyo, Japan). A Fourier transform infrared spectrometer (FTIR; Thermal Nicolet iS 50, Karlsruhe, Germany) was used. The UV–visible spectra were determined using a spectrophotometer (Hitachi U-3000, Tokyo, Japan). The Brunauer–Emmett–Teller (BET) values of the materials were measured using a nitrogen adsorption and desorption analyzer (iQinstrument Autosorb) and a thermogravimetric analyzer (Hitachi-STA 7200, Tokyo, Japan), respectively. The samples were combusted in a Thermolyne ^TM^ (Barnstead, Dubuque, IA, USA) furnace in an enclosed chamber without oxygen.

### 2.3. Preparation of Olive Stones (OS)

**Scheme 1:**
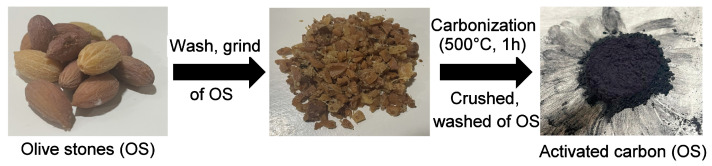
The proposed preparation pathway of OS.

The OS were extracted from olives and were washed well with water. After washing, the OS were subjected to carbonization in the absence of air at 500 °C for 1h to remove volatile components and convert the material into carbon [32]. The activated carbon was crushed and washed to remove impurities. After washing, the material was dried to obtain the final activated carbon product (Scheme 1).

### 2.4. Synthesis of Reduced Graphene Oxide (rGO)

The graphite oxide (GO) was synthesized using the developed Hummers method using graphite powder (GP) [33]. A mass of 5 g of GP was added to H_2_SO_4_ (115 mL) and NaNO_3_ (2.5 g) and stirred for 1h; then, KMnO_4_ (15 g) was slowly added and mixed at 40 °C for 1h in an oil bath. To complete the process, 10 mL of H_2_O_2_ was added to the suspension. Next, the filtered product was washed until a pH of 7.0 was reached. The washed sample (GO) was dried at 80 °C for 24 h. Then, a total of 3.7 g of GO was mixed with 60mL of KOH (8 M) and sonicated for 2 h. Then, the samples were washed and dried for 3 h at 80 °C.

### 2.5. Preparation of OS−rGO

**Scheme 2:**
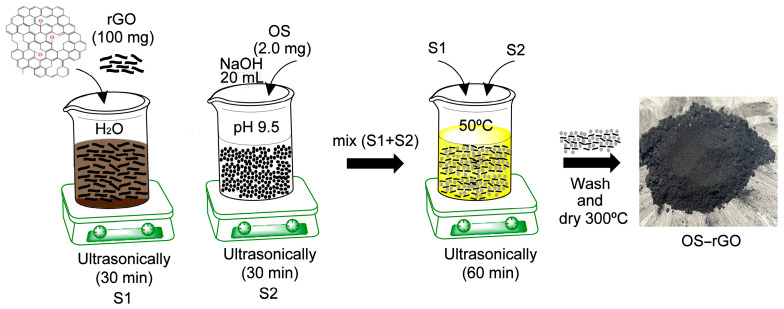
The proposed preparation pathway of OS−rGO material.

A mass of 100 mg of rGO was added to 15 mL of distilled water and dispersed for 30 min using ultrasound to obtain a negatively charged rGO suspension (S1). Separately, OS (2.0 g) were ultrasonically dispersed for 30 min in 20 mL of NaOH (pH 9.5) (S2). Next, the S1 and S2 were mixed, and the sonication time was extended for 1h at 50 °C to complete the electrostatic self-assembly process. Finally, the precipitate was collected, washed and dried at 300 °C for 1h to obtain the OS−rGO material (Scheme 2).

### 2.6. Synthesis of PAni@OS−rGO

A mass of 1.0 g of OS−rGO was added to the Ani solution in the presence of 25 mL of HCl (1 M). The functional groups on the surface of the OS−rGO facilitate the interaction with the Ani monomer, and aid in the dispersion of OS−rGO into the polymer matrix. Next, the polymerization was initiated by adding an oxidizing agent (APS) to the solution. This led to the formation of PAni chains. Simultaneously, the OS−rGO sheets became incorporated into the growing polymer structure. Next, the sediment was filtered and washed with ethanol and water, and dried for 6 h at 60 °C. The resulting PAni@OS−rGO (1:1) was stored. Likewise, the PAni@OS−rGO (2:1) material was synthesized in the same way, knowing that this electrode consisted of 2.0 g of Ani and 1.0 g of OS−rGO.

### 2.7. Electrochemical Performance

The electrochemical properties were studied using a three-electrode system (modified working electrode (WE), platinum wire counter electrode and reversible hydrogen electrode) at a fixed potential range of −0.1 V to +1.0 V in KOH (1 M) as the electrolyte at ambient temperature [7,8,9]. To prepare the WE, 70 wt% of active product, 15 wt% of CB and 15 wt% of PVDF were mixed in C₃H₆O and stirred at 60 °C until a homogeneous suspension was formed with a thickness of 100 μm. Subsequently, this suspension was drop-casted onto an SS lamina and dried overnight at 60 °C.

The specific capacitance (Csp) of the electrode was determined with Formula (1), as follows:

(1) $$\mathrm{Csp}=\frac{I\Delta t}{m}$$

The energy density (ED) and power density (PD) were determined as follows, using Formulas (2) and (3), respectively:

(2) $$\mathrm{ED}=\frac{1}{2}\mathrm{Csp}{\left(\Delta V\right)}^{2}$$

(3) $$\mathrm{PD}=\frac{E}{t}$$

where Csp (F·g^−1^) is the specific capacitance, V (V) is the potential window, *m* (g) is the active product mass, *I* (A·g^−1^) is the discharge density and t (s) is the time of discharge.

## 3. Results

### 3.1. Characterization of Samples

**Figure 1:**
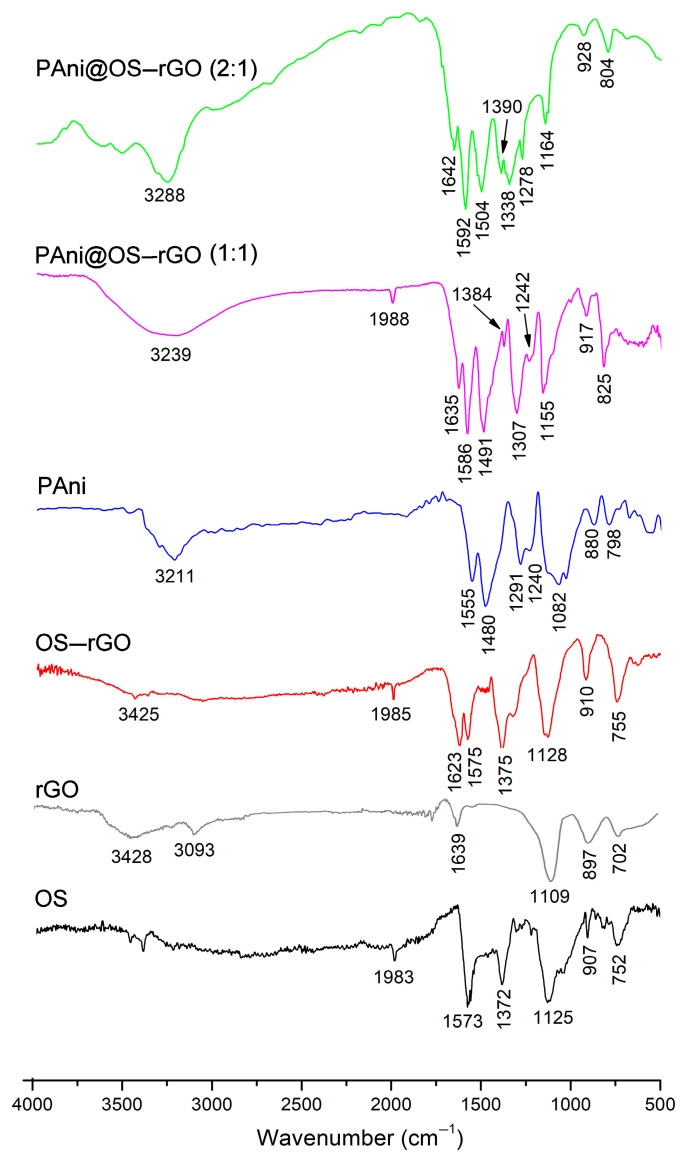
FTIR spectra of samples.

Figure 1 depicts the FTIR analyses of the synthesized OS, OS−rGO, PAni, PAni@OS−rGO (1:1) and PAni@OS−rGO (2:1). The OS spectrum shows a characteristic absorption band at 752 cm^−1^, which corresponds to the aryl C–O or the aryl C–H groups, and a band at 907 cm^−1^ corresponding to the C–H deformation of cellulose. The two bands at 1125 cm^−1^ and 1372 cm^−1^ are associated with C–O–C vibration and C–H deformation, respectively. The absorption band at 1573 cm^−1^ is attributed to the aromatic skeletal vibration in lignin. The absorption peak appearing at 1983 cm^−1^ is assigned to the C–H asymmetric stretching vibration of aliphatic CH_3_ groups [34,35]. Moreover, the formation of rGO in the OS−rGO material is clearly observed on the basis of the band at 1623 cm^−1^, ascribed to the sp^2^ structure of the C=C group [35]; moreover, all OS characteristic bands are shifted significantly to higher wavenumbers due to existence of OS–rGO bonds. This result indicates the formation of rGO sheets on the OS surface. Furthermore, the PAni shows an absorption peak at 1555 cm^−1^ that is related to C=C stretching vibrations of the quinoid units. A band at 1480 cm^−1^ represents the C–N stretching vibration adjacent to the quinoid form. The band at 1291 cm^−1^ is attributed to C–N stretching vibration of the benzenoid units. The bands near 1082 and 880 cm^−1^ represent the C–H in-plane and out-of-plane deformational vibrations of PAni, respectively. Considering the Pani@OS−rGO composites, the bands near at 917, 1155, 1384, 1635 and 1988 cm^−1^ correspond to the OS−rGO, while those at 825, 1242, 1307, 1491 and 1586 cm^−1^ correspond to the Pani matrix. It is worth mentioning that the absorption band located at 1307 cm^−1^ in the composites is due to C–N stretching vibration of the benzenoid units. The band close to 3239 cm^−1^ is attributed to N–H symmetric stretching vibration.

**Figure 2:**
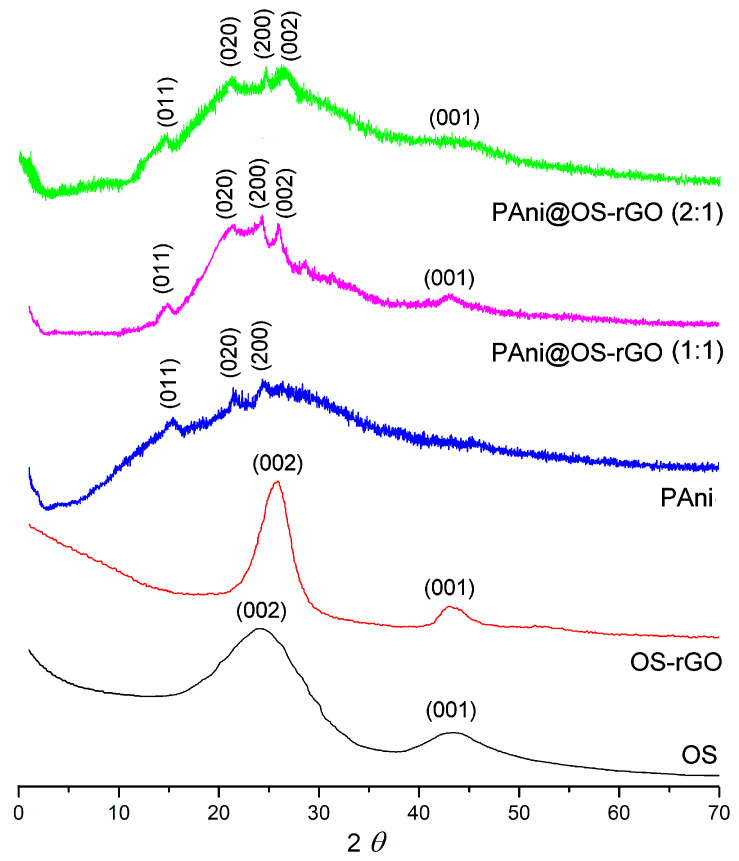
XRD patterns of samples.

Typical XRD spectra for activated carbon were observed for the studied OS (see Figure 2). The strong diffraction peak near at 2θ = 24.82° was assigned to the (002) reflection of a turbostratic carbon structure, whereas the peak at 2θ = 43.71° was attributed to the (001) plane [23]. Importantly, the (002) band of the OS−rGO is logically symmetric in profile, and corresponds to the stacking of the graphite structure basal plane. Thereby, the (002) plane located at 2θ = 25.55° indicates the presence of an ordered and disordered activated carbon structure in the OS−rGO composite [24,25]. The XRD pattern of the PAni was in accordance with the reported literature. The PAni shows diffraction peaks at 2θ values of 15.55°, 20.47°, and 24.29°, which correspond to (001), (020) and (200) planes, respectively [9]. Moreover, the XRD pattern of the PAni@OS−rGO composites shows the combined peaks of the OS−rGO composite and PAni. The peak at the (002) plane matches with the reported values for OS−rGO composite, while the peaks at the (001), (020) and (200) planes match with those of PAni.

The nanoparticle diameter of the prepared material was calculated using the following Scherrer equation:

(4) $$D=\frac{k.\lambda }{\beta cos\theta }$$

where *k =* 0.9 is the Scherrer constant and *β* is the value at FWHM (Δ2*θ* is in radian). The average crystallite sizes measured with the Scherrer formula were found to be 198 nm and 219 nm for the PAni@OS−rGO (1:1) and PAni@OS−rGO (2:1), respectively.

**Figure 3:**
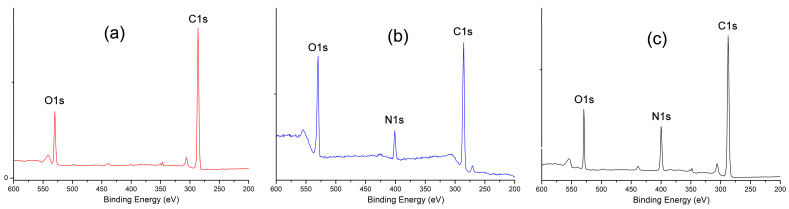
Survey scans for (**a**) OS−rGO; (**b**) PAni@OS−rGO (1:1); and (**c**) PAni@OS−rGO (2:1).

XPS analysis was employed to characterize the elemental change in the synthesized samples. Figure 3 shows the survey scan for the OS−rGO, PAni@OS−rGO (1:1) and PAni@OS−rGO (2:1). The survey scan characteristics show the existence of O and C, as well as the presence of N in the two PAni@OS−rGO samples.

**Figure 4:**
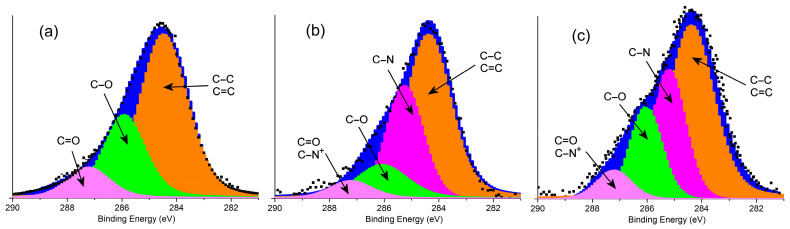
High-resolution C1s spectra for (**a**) OS−rGO; (**b**) PAni@OS−rGO (1:1); and (**c**) PAni@OS−rGO (2:1).

Figure 4 shows the C1s spectra for the OS−rGO, which deconvolute into three Gaussian peaks with binding energies of 284.49 eV (C–C/C=C), 285.95 eV (C–O) and 287.28 eV (C=O) [16]. Moreover, the C1s spectra for the PAni@OS−rGO (1:1) composite deconvolute into four Gaussian peaks at 284.38 eV, 285.26 eV, 286.12 eV and 287.33 eV, corresponding to the (C–C/C=C), (C–N), (C–O) and (C=O/C–N^+^) bonds, respectively. At the same time, the PAni@OS−rGO (2:1) also show C1s peaks at 284.34 eV (C–C/C=C), 285.21 eV (C–N), 286.05 eV (C–O) and 287.26 eV (C=O/C–N^+^).

**Figure 5:**
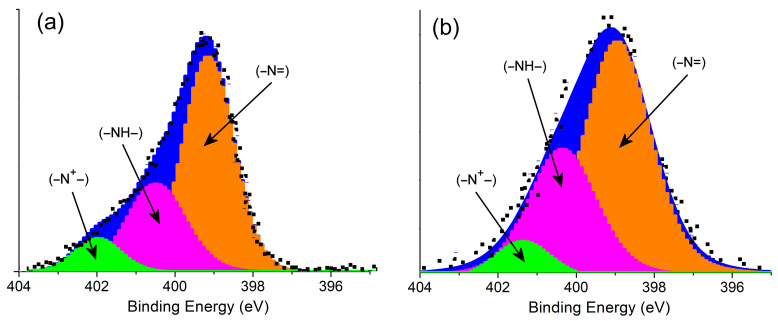
High-resolution N1s spectra for (**a**) PAni@OS−rGO (1:1) and (**b**) PAni@OS−rGO (2:1).

Figure 5 shows the high-resolution N1s spectra for the two PAni@OS−rGO composites, where they can be divided into three components. In PAni@OS−rGO (1:1) the peaks illustrated at 399.33 eV, 400.69 eV and 402.23 eV arise due to the existence of (–N=), (–NH–) and (–N^+^), respectively. The XPS spectra can be divided into three characteristic peaks, each at 398.95 eV (–N=), 400.37 eV (–NH–) and 401.39 eV (–N^+^). These characteristic peaks prove the existence of a PAni matrix on the OS−rGO surface.

**Figure 6:**
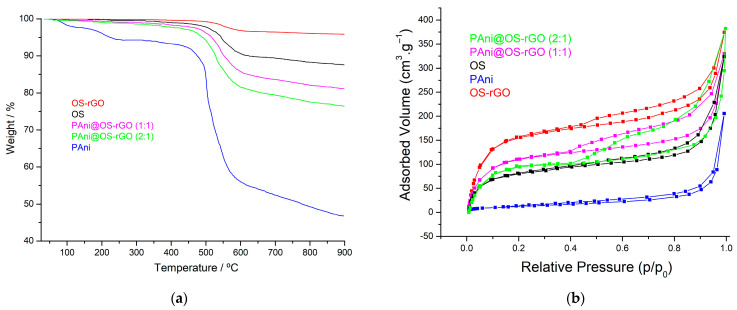
(**a**) TGA curves and (**b**) nitrogen adsorption isotherms of materials.

TGA was applied to analyze the thermal stability of the samples, as shown in Figure 6a. The two PAni@OS−rGO composites showed high stability compared to pure PAni. The OS sample is thermally stable carbon that loses very little mass on heating. Moreover, the initial weight loss from the OS is due to the loss of water, beyond which it remains stable up to 550 ℃. The PAni itself decomposed at a temperature of 120 ℃, and a sharp weight loss was observed at 180–450 °C. This could be due to the evaporation of volatile solvents and adsorbed water molecules on the surface of the prepared samples. There is a continuous decrease in weight throughout the temperature range, with a sharp decrease at 460 °C indicating the decomposition of the polymer chain. On the other hand, the TG thermogram for both PAni@OS−rGO samples shows a three-step weight loss. The first degradation step takes place up to 110 °C due to the elimination of H_2_O molecules and moisture. The second degradation step takes place in between 210 °C and 480 °C, which is mainly owing to the loss of dopant molecules. And third degradation step occurs from 500 to 900 °C, and is attributed to thermal degradation of the polymeric backbone. Furthermore, the TG curve of OS−rGO shows a much enhanced thermal stability with the least thermal degradation. Additionally, the TG thermogram of the two PAni@OS−rGO nanocomposites shows a similar thermal degradation mechanism as the OS, with increased thermal stability. This confirms the impact of OS−rGO materials on the PAni matrix, and the dependency of thermal stability on the concentration of OS−rGO composite.

Figure 6b shows the nitrogen gas adsorption/desorption isotherms plots of the materials. Type IV hysteresis loop isotherms are exhibited, which proves the presence of mesoporous materials [35]. The surface area (S_BET_) of the materials was evaluated using BET, while the pore size and the pore volume were determined using BJH analyses. It is worth mentioning that the relationship between pore texture and electrochemical properties is a critical aspect in the design and performance of energy storage devices such as supercapacitors. The pore structure of electrode materials directly influences their electrochemical behavior, including their capacitance, energy density, power density and cycling stability. According to the BET study, the OS−rGO composite possesses a higher S_BET_ of 827.5 m^2^·g^−1^ than its corresponding OS (758.2 m^2^·g^−1^), which demonstrates excellent agreement with the results obtained by Jaouadi et al. [24]. This indicates that OS was integrated into the surfaces of the rGO and supported expansion of the arrangement of sheets, thereby increasing the S_BET_ of the OS−rGO composite. However, the S_BET_ values for both the PAni@OS−rGO (1:1) (711.5 m^2^·g^−1^) and PAni@OS−rGO (2:1) (635.9 m^2^·g^−1^) were significantly reduced due to the formation of a PAni matrix (41.8 m^2^·g^−1^) on the OS−rGO surface. This can be explained by the formation of PAni into the OS−rGO surface, as the pore volumes for both the PAni@OS−rGO (1:1) (0.59 cm^3^·g^−1^) and PAni@OS−rGO (2:1) (0.51 cm^3^·g^−1^) are relatively high, whereas the OS−rGO and PAni have pore volumes of 0.62 cm^3^·g^−1^ and 0.16 cm^3^·g^−1^, respectively. Generally, the S_BET_ of the composites increases with their porosity [36,37].

### 3.2. Optical Absorption Study

**Figure 7:**
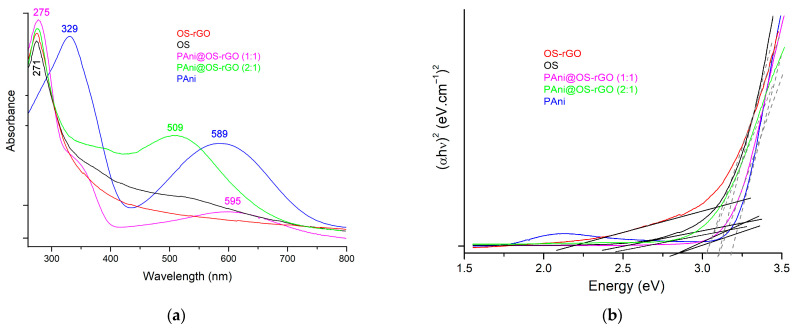
(**a**) UV–visible spectra and (**b**) Tauc plots for samples.

Figure 7a depicts the absorption spectra of the synthesized samples. The absorbance peak at 271 nm is due to the π-π∗ transition of C–C aromatic bonds in the OS, which shifted to 275 nm in the OS−rGO composite. Moreover, the PAni showed its characteristic absorbance peak at 329 nm, which is related to π-π∗ band transition; the second peak at 589 nm is caused by polaron electronic transition. When the PAni matrix is added to the OS−rGO, it produces PAni@OS−rGO materials that have lower absorption spectra. The polaron OS−rGO incorporation organizes the electronic energy when the OS−rGO interacts with the quinonoid units into the PAni. Hence, the band of PAni@OS−rGO samples shifts to the highest energy value when compared to the PAni sample.

The optical bandgap energy (*E_g_*) for the materials was determined using the following Tauc plot method (Equation (5)):

(5) $${\left(ah\nu \right)}^{n}=B\left(h\nu -E_{g}\right)$$

where hν is the bandgap energy, a is the absorption coefficient and $E_{g}$ is the energy of the photon. As illustrated in Figure 6b, graphs of ${\left(ah\nu \right)}^{2}$ vs. hν for samples are plotted.

The estimated *E_g_* values for the OS, OS−rGO, PAni, PAni@OS−rGO (1:1) and PAni@OS−rGO (2:1) were found to be 3.07, 3.03, 3.37, 3.12 and 3.16 eV, respectively (Figure 7b). When the PAni matrix was added to the OS−rGO, its $E_{g}$ increased due to a decrease in orbital overlap, since shorter bond distances usually correlate with a larger $E_{g}$ and better orbital overlap [38].

### 3.3. Electrochemical Studies

**Figure 8:**
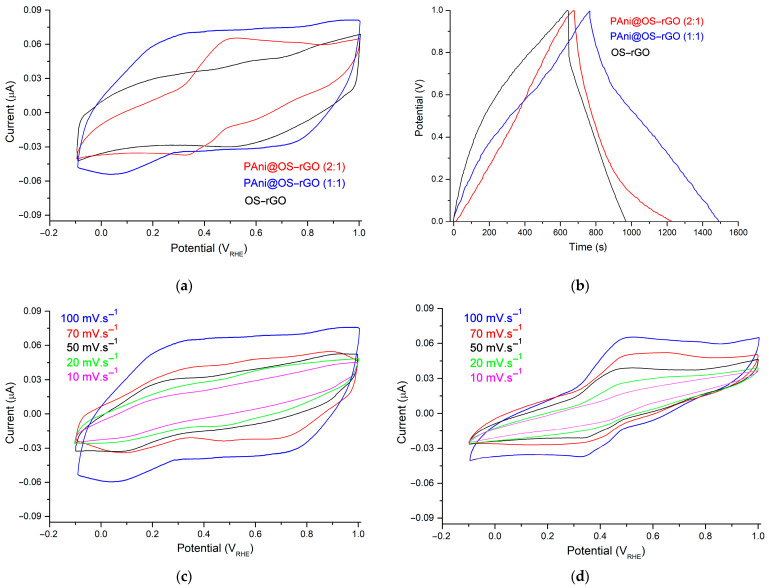
(**a**) CVs at 10 mV·s^−1^ of electrodes; (**b**) GCD of electrode materials created at 0.1 A·g^−1^; (**c**) CV curves of PAni@OS−rGO (1:1); and (**d**) CV curves of PAni@OS−rGO (2:1) at various scan rates.

The CV graph for the OS−rGO is close to a rectangle (see Figure 8a), suggesting that OS−rGO has the features of an EDLC with high reversibility [35]. The CV curve for the PAni@OS−rGO (2:1) material has a broad anodic peak near 0.49 V. However, on the reverse scan, two cathodic peak potentials at 0.58 V and 0.33 V correspond to the mutual transition between the pernigraniline–emeraldine and the emeraldine–leucoemeraldine bases of PAni. Compared to the OS−rGO and PAni@OS−rGO (2:1), the CV curve for the PAni@OS−rGO (1:1) has a higher current density peak, indicating that this electrode has a higher specific capacitance than the PAni@OS−rGO (2:1), and successfully overcomes the disadvantages of the OS−rGO’s low specific capacitance. The PAni@OS−rGO (1:1) electrode has two cathodic peaks at approximately 0.29 and 0.52 V. On the contrary, the cathodic curve displays three peaks centered at 0.71 V, 0.43 V and 0.07 V, due to the transition between the oxidized/reduced forms of PAni in this hybrid material. Additionally, the PAni@OS−rGO (1:1) displayed a maximum CV area, suggesting an excellent capacitive behavior and the highest charge storage capability [39]. This characteristic was confirmed by comparison with the constant current charge and discharge (GCD) graphs exhibited in Figure 8b. Measured from the GCD graphs for electrodes at 0.1 A·g^−1^, the PAni@OS−rGO (1:1) shows the highest specific capacitance of 582.6 F·g^−1^, which is better than the PAni@OS−rGO (2:1) (453.3 F·g^−1^) and the OS−rGO (524.8 F·g^−1^). Furthermore, the GCD curves for these materials were triangular in form, and no significant voltage drop (IR) was remarked, further confirming the good charge storage characteristics of these electrodes. In addition, Figure 8c shows that the shape of the CVs for the PAni@OS−rGO (1:1) electrode is almost rectangular at various scan rates, which is typical of capacitive behavior due to reduced ion mobility and high internal resistance. Moreover, the current density progressively increases with the increasing scan rate, suggesting that it has excellent electrochemical responsiveness. On the other hand, the CV curves in Figure 8d related to the PAni@OS−rGO (2:1) present an oxidation peak near 0.49 V, with two reduction peaks at 0.58 V and 0.33 V in the windows ranging from −0.1 V to +1.0 V at scan speeds between 10 mV·s^−1^ and 100 mV·s^−1^. These suggest that redox pseudo-capacitances were the predominant capacitance form. Even at 100 mV·s^−1^, the redox characteristic is preserved with only a slight move, proving a high capacity.

In other words, the (1:1) ratio shows that the loading of PAni on the OS−rGO is moderate, resulting in a well-dispersed distribution of polymer on the OS−rGO surface. Furthermore, the conductive nature of rGO may dominate the electrical conductivity of the composite due to the moderate loading of PAni. This impacts the overall electrochemical conductivity of the material. In addition, the combination of PAni and OS−rGO in a balanced ratio leads to synergistic effects, where the strengths of both materials contribute to enhanced electrochemical performance, resulting in improved capacitance and charge storage capabilities. Otherwise, the (2:1) ratio means that there is a higher loading of PAni on the OS−rGO. This increased loading may affect the distribution of the polymer on the OS−rGO surface. While a higher PAni loading may offer additional redox-active sites, there is a risk of diminishing its synergistic effects with the OS−rGO. Achieving a balanced ratio is crucial for maximizing the benefits of both components.

**Figure 9:**
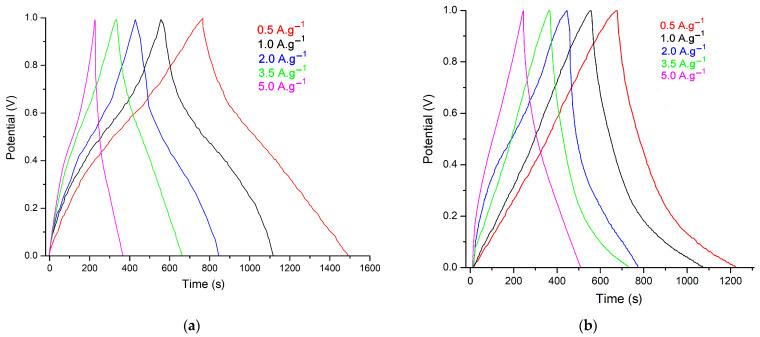

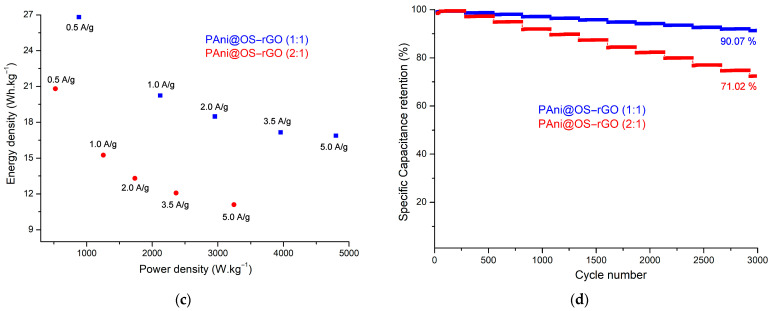
(**a**) GCD of PAni@OS−rGO (1:1); (**b**) GCD of PAni@OS−rGO (2:1) at scan rate of 10 mV·s^−1^ and different current densities; (**c**) Ragone plots for electrodes at various current densities; and (**d**) rate performance test for 3000 consecutive cycles at 0.5 A·g^−1^.

A comparison of the GCD for the PAni@OS−rGO (1:1) and PAni@OS−rGO (2:1) electrodes at different current densities is shown in Figure 9a,b, respectively. The GCD of these electrodes depict a quasi-triangular shape of the galvanostatic GCD, which are not completely straight lines, implying that a Faradaic reaction often occurred, and confirming the pseudo-capacitive behavior in the electrodes over the range of current densities. Furthermore, Figure 9c also represents the relationship between energy density and power density, which is a non-linear relationship that becomes stable at higher current densities. The energy density and power density of the PAni@OS−rGO (1:1) were found to be 26.82 Wh·Kg^−1^ and 882 W·kg^−1^, respectively, which is better than the values for the PAni@OS−rGO (2:1) (20.55 Wh·Kg^−1^ and 522 W·kg^−1^, respectively). The higher energy density can be attributed to the interconnected network of the PAni@OS−rGO, particularly for the PAni@OS−rGO (1:1) electrode (26.82 Wh·Kg^−1^), due to the conducting highway illustrated in the Faradaic characteristic and charge transport. Also, the specific capacitance retention was found to be 90.07% and 71.02% for 3000 consecutive cycles at 0.5 A·g^−1^ for the PAni@OS−rGO (1:1) and PAni@OS−rGO (2:1) electrodes, respectively (Figure 9d). The electrodes in this research are comparable to those in other previous reports [7,13,40,41,42,43,44,45,46,47,48,49]; these results suggest the superiority of PAni@OS−rGO materials over those of other rGO-based electrodes (see Table 1).

**Figure 10:**
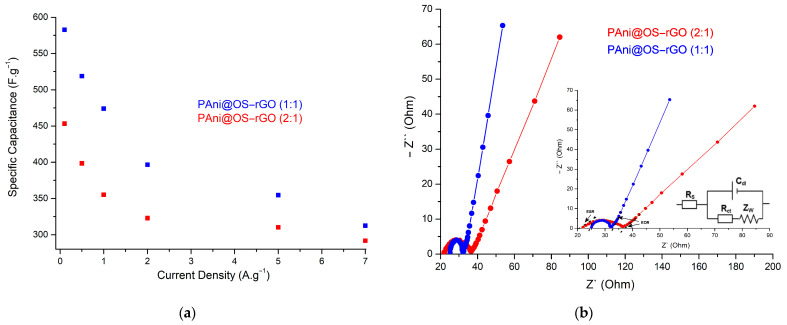
(**a**) Variations in specific capacitances of electrodes at various current densities; and (**b**) Nyquist plots for electrode materials (inset: model of Randles equivalent circuit).

The specific capacitance values of the two electrodes gradually increase from 0.1 A·g^−1^ to 7.0 A·g^−1^ (Figure 10a), exhibiting good rate performance, which is assigned to their unique interbonded structures and rationalistic hierarchal porous structures. At 0.1 A·g^−1^, the yielded specific capacitances are 582.6 F·g^−1^ and 453.3 F·g^−1^, and the corresponding capacitance retentions are 53.64% and 64.37%, obtained at 7.0 A·g^−1^ for PAni@OS−rGO (1:1) and PAni@OS−rGO (2:1), respectively.

In addition, electrochemical impedance spectroscopy (EIS) analysis permits us to comprehend charge transfer and its kinetics. Therefore, it is necessary to subject the synthesized electrodes to EIS study. A typical EIS is characterized by a low-frequency region and a high-frequency region. A high-frequency region describes a semicircle and indicates the charge transfer resistance (R_ct_). In principle, it indicates the resistance to the charge to attain the OS−rGO through the PAni matrix. As the current passes, the PAni matrix suffers from the resistance of the solution and R_ct_. Generally, both R_s_ and R_ct_ must be as low as possible, since ion mobility determines the electrochemical performance. As exhibited in Figure 10b, the PAni@OS−rGO (1:1) presents a low R_s_ and R_ct_ of 4.92 Ω and 1.9 Ω, respectively, in comparison with values for the PAni@OS−rGO (2:1) (5.77 Ω and 3.2 Ω, respectively). These lower values can be attributed to the interconnected PAni matrix-decorated OS−rGO sheets. Moreover, the R_ct_ of the PAni@OS−rGO (1:1) electrode was reduced compared with the PAni@OS−rGO (2:1), indicating that the participation of PAni with OS−rGO at a 1:1 ratio can accelerate electron exchange on the material surface. Furthermore, the values of equivalent series resistance (ESR) recorded from the semicircle at the high-frequency region are 24.8 Ω and 20.7 Ω for the PAni@OS−rGO (1:1) and PAni@OS−rGO (2:1) electrodes, respectively.

## 4. Conclusions

In summary, novel PAni@OS−rGO electrodes were designed and assembled via OS−rGO preparation and subsequent in situ polymerization of aniline (PAni to OS−rGO ratios of (1:1) and (2:1)). XRD, FTIR, UV–vis and XPS analyses showed that the PAni matrix is distributed around the OS−rGO sheets. TGA and BET analyses showed that the synthesized electrodes have a higher S_BET_ and stability. The obtained electrodes exhibit excellent electrochemical performance in energy storage. Based on the structure and performance study, the integration of OS−rGO can not only increase the electrode conductivity, but it also ameliorates PAni cycling stability. The specific capacitance of the PAni@OS−rGO (1:1) reached 582.6 F·g^−1^ at 0.1 A·g^−1^, while the specific capacitance was still 312.5 F·g^−1^ at 7.0 A·g^−1^ with an excellent retention of 53.64%. Excellent cycling stability with a capacity retention of 90.07% after 3000 cycles further proves that the material is a promising electrode for SCs. The low cost, mass production potential and the easy fabrication of the PAni@OS-rGO material are instrumental to the expected synthesis of a prototype of this supercapacitor for battery and other energy storage device applications.

## Figures and Tables

**Table 1:** Electrochemical performances of various electrode materials.

Electrode Materials	Specific Capacitance (F·g^–1^)	Energy Density (Wh·kg^–1^)	Power Density (W·kg^–1^)	Reference
PEDOT@WO_3_–GO	478.3	54.2	971	[7]
Graphene/Fe_2_O_3_	378.7	64.09	800.01	[13]
CWCC-rGO@PVA	288	36	3600	[40]
rGO/PANI/urchin-like mesoporous TiO_2_	464	34	3720	[41]
rGO/Poly(1,5 dihydroxynaphthalene)/TiO_2_	556	//	//	[42]
Ti3C2TX@PANI-rGO	617.84	33	503.42	[43]
PANI–rGO–MWNTs	498	//	//	[44]
rGO-CNT-PANI	741	92.4	6300	[45]
α-MnO_2_/PANI/rGO	661	11	1250	[46]
N-doped rGO/PANI	510	24.7	329.5	[47]
rGO/MoS_2_/PANI	160	22.3	5080	[48]
rGO/UCNTs/PANI	53	7.4	189	[49]
Fe_3_Mo_3_C/Mo_2_C-800	202.3	39.9	1800	[50]
SrCo_0.9_Fe_0.1_O_3-δ_	1035.9	26.2	800	[51]
PPy700@SFO@CC	421	16.9	984	[52]
Cu_2_MoS_4_	152.6	16.8	800	[53]
PAni@OS–rGO (1:1)	582.6	26.82	882	This study
PAni@OS–rGO (2:1)	453.3	20.55	522	This study

## Data Availability

Data are contained within the article.

## Supplementary Materials

The following supporting information can be downloaded at: https://www.mdpi.com/article/10.3390/polym16030421/s1. Table S1: The performance of graphene and PAni composites for supercapacitors. Refs. [54,55,56,57] are cited in the Supplementary Materials.
