# Supplementary material for: Investigation of Hybrid Electrodes of Polyaniline and Reduced Graphene Oxide with Bio-Waste-Derived Activated Carbon for Supercapacitor Applications
Source: Polymers (Basel). 2024 Feb 2;16(3):421. doi: 10.3390/polym16030421 (PMC10857362; doi:10.3390/polym16030421)
Supplement: Supplementary file 1 [file polymers-16-00421-s001.zip › polymers-2829078-supplementary.pdf]

polymers

MDPI

Supplementary Materials

Investigation of Hybrid Electrodes of Polyaniline and Reduced Graphene Oxide with Bio-Waste-Derived Activated Carbon for Supercapacitor Applications

Imen Benchikh [1], Abdelrahman Osama Ezzat [2], Lilia Sabantina [3,4,*], Youcef Benmimoun [5] and Abdelghani Benyoucef [5,*]

[1] Faculty of Science, University of Amar Telidji Laghouat, Laghouat 03000, Algeria; i.benchikh@lagh-univ.com
[2] Department of Chemistry, College of Sciences, King Saud University, Riyadh 11451, Saudi Arabia; aezzat@ksu.edu.sa
[3] Department of Apparel Engineering and Textile Processing, Berlin University of Applied Sciences-HTW Berlin, 12459 Berlin, Germany
[4] Department of Textile and Paper Engineering, Polytechnic University of Valencia (UPV), 03801 Alcoy, Spain
[5] Water Science and Technology Laboratory, University of Mustapha Stambouli Mascara, Mascara 29000, Algeria; youcef.benmimoun@univ-mascara.dz
* Correspondence: Correspondence: lilia.sabantina@htw-berlin.de (L.S.); a.benyoucel@univ-mascara.dz (A.B.)

Table S1. The performance of graphene and PAni composites for supercapacitors.

Electrode Materials (3-electrode)	Specific Capacitance ($F{\cdot}g^{-1}$)	Current Densities ($A{\cdot}g^{-1}$)	Cycle Life	References
PEDOT@WO_3–GO	478.3	0.1	92.1% capacitance after 5000 cycles (50 $mV{\cdot}s^{-1}$)	[1]
Grapheme/PAni	233	0.1	//	[2]
rGO/PAni	464	0.62	13.5% capacitance after 2000 cycles (50 $mV{\cdot}s^{-1}$)	[3]
GO/PAni	475	5.0	90% capacitance after 2000 cycles (10 $mV{\cdot}s^{-1}$)	[4]
PAni/GO	442	1.0	83% capacitance after 2000 cycles (2 $mV{\cdot}s^{-1}$)	[5]
UGA/PANI	538	1.0	74% capacitance after 1000 cycles (50 $mV{\cdot}s^{-1}$)	[6]
GO/PG/PAni	793.7	1.0	80% capacitance after 1000 cycles (100 $mV{\cdot}s^{-1}$)	[7]

References

1. Memou, C.H.; Bekhti, M.A.; Kiari, M.; Benyoucef, A.; Alelyani, M.; Alqahtani, M.S.; Alshihri, A.A.; Bakkour, Y. Fabrication and Characterization of a Poly(3,4-ethylenedioxythiophene)@Tungsten Trioxide–Graphene Oxide Hybrid Electrode Nanocomposite for Supercapacitor Applications. *Nanomaterials*. **2023**, *13*, 2664; https://doi.org/10.3390/nano13192664.
2. Wang, D.W.; Li, F.; Zhao, J.; Ren, W.; Chen, Z.H.; Tan, J.; Wu, Z.S.; Gentle, I.; Lu, G.Q.; Cheng, H.M. Fabrication of Graphene/Polyaniline Composite Paper viaIn Situ Anodic Electropolymerization for High-Performance Flexible Electrode. *ACS Nano*. **2009**, *3*, 1745-1752. https://doi.org/10.1021/nn900297m.
3. Wang, H.; Li Ma, Gan, M.; Zhou, T.; Sun, X.; Dai, W.; Wang, H.; Wang, S. Design and assembly of reduced graphene oxide/polyaniline/urchin-like mesoporous TiO_2 spheres ternary composite and its application in supercapacitors. *Composites Part B: Engineering*. **2016**, *92*, 405-412. https://doi.org/10.1016/j.compositesb.2016.02.047.
4. Chang, T.W.; Lin, L.Y.; Peng, P.W.; Zhang, Y.X.; Huang, Y.Y. Enhanced electrocapacitive performance for the supercapacitor with tube-like polyaniline and graphene oxide composites. *Electrochim. Acta*. **2018**, *259*, 348-354. https://doi.org/10.1016/j.electacta.2017.10.195.

5. Li, Z.F.; Zhang, H.; Liu, Q.; Liu, Y.; Stanciu, L.; Xie, J. Covalently-grafted polyaniline on graphene oxide sheets for high performance electrochemical supercapacitors. *Carbon*. **2014**, *71*, 257-267. https://doi.org/10.1016/j.carbon.2014.01.037.
6. Wu, X.; Tang, L.; Zheng, S.; Huang, Y.; Yang, J.; Liu, Z.; Yang, W.; Yang, M. Hierarchical unidirectional graphene aerogel/polyaniline composite for high performance supercapacitors. *J. Power Sources*. **2018**, *397*, 189-195. https://doi.org/10.1016/j.jpowsour.2018.07.031.
7. Zhang, Y.; Si, L.; Zhou, B.; Zhao, B.; Zhu, Y.; Zhu, L.; Jiang, X. Synthesis of novel graphene oxide/pristine graphene/polyaniline ternary composites and application to supercapacitor. *Chem. Eng. J*. **2016**, *288*, 689-700. https://doi.org/10.1016/j.cej.2015.12.058.

Disclaimer/Publisher's Note: The statements, opinions and data contained in all publications are solely those of the individual author(s) and contributor(s) and not of MDPI and/or the editor(s). MDPI and/or the editor(s) disclaim responsibility for any injury to people or property resulting from any ideas, methods, instructions or products referred to in the content.
